# Fabrication and Characterization of Spongy Denuded Amniotic Membrane Based Scaffold for Tissue Engineering

**DOI:** 10.22074/cellj.2015.493

**Published:** 2015-01-13

**Authors:** Ehsan Taghiabadi, Sima Nasri, Saeed Shafieyan, Sasan Jalili Firoozinezhad, Nasser Aghdami

**Affiliations:** 1 Department of Biology, Faculty of Science, Payame NOOR University, Tehran, Iran; 2 Department of Regenerative Biomedicine at Cell Science Research Center, Royan Institute for Stem Cell Biology and Technology, ACECR, Tehran, Iran

**Keywords:** Extracellular Matrix, Skin Substitute, Biodegradable

## Abstract

**Objective:**

As a biological tissue material, amniotic membrane (AM) has low immunogenicity and to date has been widely adopted in clinical practice. However, some features such as low biomechanical consistency and rapid biodegradation is limited the application of AM. Therefore, in this study, we fabricated a novel three-dimensional (3D) spongy scaffold made of the extracellular matrix (ECM) of denuded AM. Due to their unique characteristics which are similar to the skin, these scaffolds can be considered as an alternative option in skin tissue engineering.

**Materials and Methods:**

In this experimental study, cellular components of human amniotic membrane (HAM) were removed with 0.03% (w/v) sodium dodecyl sulphate (SDS). Quantitative analysis was performed to determine levels of Glycosaminoglycans (GAGs), collagen, and deoxyribonucleic acid (DNA). To increase the low efficiency and purity of the ECM component, especially collagen and GAG, we applied an acid solubilization procedure hydrochloridric acid (HCl 0.1 M) with pepsin (1 mg/ml). In the present experiment 1-ethyl-3-(3-dimethyl aminopropyl) carbodiimide hydrochloride (EDC)/N-hydroxysuccinimide (NHS) cross linker agent was used to improve the mechanical properties of 3D lyophilized AM scaffold. The spongy 3D AM scaffolds were specified, by scanning electron microscopy, hematoxylin and eosin (H&E) staining, a swelling test, and mechanical strength and *in vitro* biodegradation tests. Human fetal fibroblast culture systems were used to establish that the scaffolds were cytocompatible.

**Results:**

Histological analysis of treated human AM showed impressive removal of cellular components. DNA content was diminished after treatment (39 ± 4.06 μg/ml vs. 341 ± 29.60 μg/ml). Differences were observed between cellular and denude AM in matrix collagen (478 ± 18.06 μg/mg vs. 361 ± 27.47 μg/mg).With the optimum concentration of 1 mM NHS/EDC ratio1:4, chemical cross-linker agent could significantly increase the mechanical property, and resistance to collagenase digestion. The results of 2, 4, 6-Trinitrobenzenesulfonic acid (TNBS) test showed that cross-linking efficiency of AM derived ECM scaffolds was about 65% ± 10.53. Scaffolds treated with NHS/EDC cross-linker agent by 100 μg/ml collagenase, lost 75% of their dry weight after 14 days. The average pore size of 3D spongy scaffold was 160 µm measured from scanning electron microscope (SEM) images that it is suitable for cell penetration, nutrients and gas change. In addition, the NHS/ EDC cross-linked AM scaffolds were able to support human fetal fibroblast cell proliferation *in vitro*. Extracts and contact prepared from the 3D spongy scaffold of AM showed a significant increase in the attachment and proliferation of the human fetal fibroblasts cells.

**Conclusion:**

The extra-cellular matrix of denuded AM-based scaffold displays the main properties required for substitute skin including natural *in vitro* biodegradation, similar physical and mechanical characterization, nontoxic biomaterial and no toxic effect on cell attachment and cell proliferation.

## Introduction

The skin, which is the largest tissue in human body, is constructed of three layers epidermis, dermis and hypodermis. It performs a main function in protecting the human body from much chemical and mechanical damage from the surrounding environment. The loss of skin can occur for various reasons, such as thermal trauma, genetic disorders, chronic wounds, burns or even surgical interventions (1). Because of the low immunogenicity of donor skin and the limited availability of donor skin sources, skin grafts are unable to provide complete recovery of the skin rendering them unsuitable for widespread use. Several years ago, great attempts were made to fabricate substitute human skin. Tissue engineering is an impressive way to develop skin substitutes and improve the wound healing. One fundamental purpose of cell biologists in tissue engineering is to induce cell proliferation using appropriate cell culture conditions (2, 3). The aim of study to construct a novel three-dimensional (3D) scaffold that improves cell proliferation and imitates the structure of the skin. Collagen is a main component of human connective tissues, particularly in soft skin tissues (4). Collagen is one of the best biomaterials for different applications (5, 6). Advantages include excellent biocompatibility, low toxicity and excellent biodegradability (7, 8). Despite these advantages, low mechanical consistency and the fast degradation rate of uncross-linked natural scaffolds such as collagen are the main problems that restrict its application. Thus, cross-linking of the collagen scaffolds is an impressive procedure to optimize their mechanical strength and to regulate the biodegradation rate. Glycosaminoglycans (GAGs) are another principal component of the skin tissue that is associated with skin repair. Being the most hydrophilic molecule exist in the natural tissue such as skin, this characteristic GAGs is of vital role in water maintenance (9). GAGs can be better modified by functional groups such as hydroxyl and carboxyl chains (10). GAGs also perform general functions in intermediary skin-cell biological procedures, cell migration, growth, granulation tissue matrix formation, inflammatory response temperance, re-epithelialization and scarring (11). In addition, cellular attachment, growth and differentiation depends on denatured collagen structure.

Human amniotic membrane (HAM) has alots of specifications that cause it applicable biomaterial. The extra cellular matrix of amnion composed of collagen I, collagen III, collagen IV and fibronectin. It is cheap and quickly takes, and its availability is virtually unrestricted. HAM has been presented to consult applicable wound protection and have a symbolic outcome on pain reduction (12). 3D natural and synthetic scaffolds play a main function in maintenance of cell proliferation and tissue regeneration (13). Interactions between cells and the extracellular matrix (ECM) are responsible for the control of cell action. So, cells grown in a 2D monolayer cannot cope with the relative complexity of the *in vivo* micro-environment. For example, it has been suggested that cells cultivated on 2D layer such as culture plates, lose numerous critical signals, important regulators, and tissue phenotypes. Cells growing in 3D have different propagation capacity, extracellular matrix synthesis, cell congestion, and metabolic functions (13).

In general, easily degradation components of AM causesa loss of mechanical property (14). So, our hypothesize in this study, are cross-linking ECM components may change the degradation rate and biomechanical specifications, thus improving their biocompatibility. The procedure of cross-linking natural scaffolds is the best method for improving the mechanical features. Commonly, there are two different technique for this purpose: physical procedure and chemical treatment (14, 15). Chemical cross-linking procedure is an appropriate method compared with physical procedure because of high mechanical strength and biodegradation rate (16). 1-ethyl-3-(3-dimethyl aminopropyl) carbodiimide hydrochloride (EDC)/N-hydroxysuccinimide (NHS) is great interest and zero-length cross-linking agent because of two different reactive groups that are able straightly join 2 different amino acid side chains (15, 16). The cross-linking of bio-scaffolds has become one of the most suitable strategies for the bio-porous matrix. Commonly, there are two types of cross-linking methods often applied in improving the mechanical properties: physical treatments and chemical techniques (14, 15). Physical treatments generally cannot output a high enough cross-linking degree to meet the demands for mechanical strength and biodegradation rates, therefore, treatments by chemical techniques are still essential in most cases (16). A cross-linking agent, EDC/NHS is of great interest in maximizing the extent of cross-linking because it contains 2 different reactive groups that are able to directly link 2 various amino acid side chains, and it is a zero-length cross-linking agent (15, 16). Therefore, we fabricated 3D spongy scaffold derived amniotic membrane (AM) specially collagen component with chemical cross-linker NHS/EDC. The porosity of sponge-like scaffold was assessed by *in vitro* cultured of human fetal fibroblasts (FBs).

## Materials and Methods

### Harvest and preparation of HAMs

In this experimental study, after written informed consent was obtained, human placentas were taken from HAMs bank, part of the public cord blood bank in the Royan Institute, with Ethical Committee Approval. All placenta donors were serologically negative for human immunodeficiency virus, hepatitis virus type B, hepatitis virus type C, and syphilis.

The placentas were washed 3 times by phosphate- buffered saline (PBS, pH=7.4, Gibco, USA) in a class 2 laminar flow. After separation of AM from the underlying chorionand cut into pieces of approximately 5×5 cm^2^. The pieces were stored in PBS containing 1.5% dimethyl sulfoxide (DMSO) at -70˚C for up to five months.

### Decellularization of HAM

The HAM was thawed then rinsed 3 times with PBS (Gibco, USA) and then incubated in hypotonic tris buffer (10 mM tris) (Merck, Germany), pH=8.0 including ethylenediaminetetraacetic acid (EDTA, 0.1% w/v) (Sigma, USA) at 4˚C for 16 hours. The AM was then put in 0.03% (w/v) solution sodium dodecyl sulphate (SDS) (Merck, Germany) in tris-buffered saline (TBS) (Sigma, USA) containing EDTA (0.1% w/v, pH=7.6) and shaken at room temperature for 24 hours. In the next step, the AM was washed in TBS (pH=7.6). The AM was incubated in a buffer contain [50 mM tris hydrochloric acid (HCl), 10 mM magnesium chloride], pH=7.5, (Sigma, USA) for 3 hours at 37˚C, on the shaker, then rinsed 3 times with PBS (Gibco, USA) (17).

### DNA quantitative assay

A DNA quantitative assay was undertaken in five denuded AM samples selected randomly, with total DNA extracted using a DNA assay kit (Roche, Germany) according to the manufacturer’s instructions. Optical density (OD) was measured at 260 nm with a micro-plate fluorescence reader (Thermo, USA). A standard curve was mapped to calculate the DNA concentration. Intact AM was used as the control.

### Manufacturing AM spongy scaffold

A solution of HCl 0.1 M, pepsin and freeze dried powder of acellular HAM were mixed to a final concentration of, 1 mg/ml, and, respectively. The mixed solution was added into a 24 wells and frozen at −70˚C for 24 hours. The scaffold size could be adjusted by (regulating) the appropriate volume of the (constructing) solution. The sponge AM scaffold was fabricated by lyophilizing for 24 hours (18). The procedure of cross-link was done for 24 hours at 25˚C in ethanol 95% (Merck, Germany) containing 1 mM NHS/EDC (Sigma, USA) with a ratio of 1:4. Afterwards, the cross-linking reaction was stopped by elimination of NHS/EDC solution and adding with 0.1 M Na_2_HPO_4_ solution then washing with distilled H_2_O more three times remove un-reacted chemicals. The scaffold was lyophilized for another 24 hours and sterilized by ethanol 70% (Merck, Germany).

### Histology and microscopy

Cellular AM, acellular AM and 3D spongy scaffold for light microscopy were fixed using 10% (w/v) neutral-buffered formalin (Sigma, USA) dehydrated and embedded in paraffin wax. Sections were cut using a microtome at 6 μm and stained with hematoxylin and eosin (H&E), collagen, GAGs and Russell-Movat stain. All histological sections were viewed using an olympus BX71 light microscope (Olympus, Germany).

### Collagen analysis

An estimation of the collagen content of the experimental groups including intact AM, denuded AM and 3D spongy AM scaffold was made by determining the hydroxyproline content in acidhydrolyzed samples by acid/pepsin-soluble Sicrol collagen assay kit (Biocolor, UK) according to the manufacturer’s instruction. For extraction of acid/ pepsin soluble collagen, samples were digested with 0.5 M acetic acid containing 1 mg/ml (w/v) pepsin (Sigma, USA) overnight at 4˚C. The supernatant of digested suspension was incubated with 1 mL Sircol dye reagent for 30 minutes at room temperature. Hydroxyproline levels were obtained by measuring absorbance at 555 nm. All contents were normalized with 0.5 mg of dry AM.

### GAG analysis

The GAG content of acid-hydrolyzed experimental groups was determined using sulfated GAG kit (Biocolor, UK) according to the manufacturer’s instruction (19, 20). GAG levels were obtained by measuring absorbance at 656 nm and extrapolating values from a standard curve of chondroitin sulphate B (Blyscan, UK). Data is expressed as μg/ mg of AM groups.

### Determination of extent of cross-linking

The 2, 4, 6-trinitrobenzenesulfonic acid (TNBS) assay was used to determine the amount of free amino groups in each of the experimental AM groups. The test samples were weighed and reacted with 0.5 ml of a 4% (w/v) NaHCO_3_ solution and 0.5 ml of a freshly made solution of 0.05% (w/v) TNBS. After reaction for 2 hours at 40˚C, 1.5 ml of 6 M HC1 was added and the samples were hydrolyzed at 60˚C for 90 minutes. The reaction mixture was diluted with distilled water (2.5 ml), cooled to room temperature and the absorbance at 420 nm was measured using a microplate fluorescence reader (Thermo, USA). Controls (blank samples) were prepared using the same procedure, except that HCl was added prior to the TNBS solution. The absorbance of the blank samples was subtracted from each sample absorbance. The absorbance was correlated to the concentration of free amino groups using a calibration curve obtained with glycine in an aqueous NaHCO_3_ solution (0.1 mg/ml), where the relationship between absorbance and concentration of primary amino groups was expressed as percent. The extent of cross-linking of 3D spongy scaffold was calculated using the following equation (21). Results were the average of five independent measurements.

$$\mathrm{Cross-linking degree (\%)}=\frac{\mathrm{Absorbance of crosslinked scaffold}}{\mathrm{Absorbance of uncrosslinked scaffold}}$$

### Scanning electron microscopy

After fixation of scaffolds in 2.5% glutaraldehyde and 0.1 M sodium phosphate buffer, pH=7.2 for 12 hours , scaffolds were immersed in 1% osmium tetroxide for 1 hour, dehydrated in ethanol and dried. Then, the scaffolds were subjected to scanning electron microscopy (18). For scanning electron microscope (SEM), the 3D spongy AM scaffold was further dried with carbon dioxide in a critical point dryer (Balzers, Liechtenstein) and coated with gold in a sputter coater (Hitachi, Tokyo, Japan) before examination under a KYKYEM3200, Germany, SEM with an accelerating voltage of 24 kV. For assessment average pore size of scaffolds, the poresize of 30 pores on each of the 5 SEM photos were measured.

### In vitro collagenase degradation

Collagenase cleaves triple helical collagen at a specific site of chains (Gly 775-Leu/Ile 776). Dry spongy scaffolds (40 mg/ml) were floated at 6 well cell culture containing 1 ml of PBS buffer (pH=7.4, Gibco, USA) with collagenase (100 μg/ml) for 3, 7, 14, and 21 days. A reaction was incubated at 37˚C. At the end of the test periods, the weight of residual dry spongy scaffolds was assessed following three times washing in distilled water and finally lyophilization. The rate of degradation was calculated from the weight of the remaining scaffold. The control group was untreated scaffold.

### PBS swelling property

The water absorption capacity of the AM scaffolds were specified by swelling scaffolds in PBS at 37˚C, pH=7.4. The scaffolds with (40 mg) were separately immersed into PBS solution for 5 minutes, 1, 2, 3, 4, 5, 6, 24, 48 and 72 hours. The water of scaffolds were re moved and then weighed. For calculation of water absorption in the swollen scaffold was used of this equation:

$$\mathrm{Water absorption}=\frac{\mathrm{Ww}-\mathrm{Wd}}{\mathrm{Wd}}$$

Ww=weight of the swollen scaffold, and Wd =weight of the dry scaffold (18).

### Cell viability assay-MTS test

MTS assessment applied in this study for evaluation of cell viabilities and proliferation rate (22). Fetal fibroblasts (104 cells/well) were seeded in 96-well plates for 72 hours at 37˚C. After 72 hours complete culture medium was removed. Then 200 μl of Culture medium and MTS solution (Promega, USA) in a proportion of 5:1 were exposed for 3 hours at 37˚C in a humidified atmosphere containing 5% CO_2_. After this time, absorbance was measured at 490 nm with a plate reader (Thermo, USA).

### Biocompatibility of 3D spongy HAM scaffold

Human fetal fibroblast proliferation and cell metabolic activities in scaffolds was assessed by measuring the mitochondrial dehydrogenase activity using 3-(4, 5-dimethylthiazol-2-yl)-5-(3- carboxymethoxyphenyl)-2-(4-sulfophenyl)-2Htetrazolium (MTS) assay (Sigma, USA), according to the manufacturer’s protocol. Scaffolds were located in 24-wells plates. Cells were seeded on the scaffolds (105 cells in 400 ml per well) and cultured for 3, 7, 14 and 21 days at 37˚C/ 5% CO_2_ in fibroblast medium (10% (v/v) fetal bovine serum, 4 mM glutamine, penicillin (100 U/ml) and streptomycin (100 mg/ml) in Dulbeccoʼs Modified Eagle Medium: Nutrient Mixture F-12 (DMEM/ F12). After the addition of 20 μl MTS per well and subsequent incubation for 3 hours at 37˚C, 5% CO_2_, the absorption was measured at 490 nm (23).

### Cell morphology

Cell morphology was distinguished using SEM. 15 mm scaffolds were put in 24-well culture dishes and cells were seeded on top. Human fetal fibroblasts were cultivated on the airside of the scaffolds with a density of about 105 cells per cm^2^ in fibroblast medium (10% (v/v) fetal bovine serum, 4 mM glutamine, penicillin (100 U/ml), and streptomycin (100 mg/ml) in DMEM/F12. Cells were cultivated at 37˚C and 5% CO_2_ for 7 days. After 7 days, scaffolds were fixed by immersion in 2% (v/v) glutaraldehyde in 0.1% osmium tetroxide for 1 hour, dehydrated in ethanol and dried. Then, the scaffolds were subjected to scanning electron microscopy. At every indicated time interval (3, 7, 14 and 21 days), the scaffolds were collected for experimental analysis.

### Cell metabolic activities in scaffolds

Cells in scaffolds were quantitatively evaluated with MTS assay at 3, 7, 14 and 21 days. 100 μl of culture medium was aspirated at 3, 7, 14 and 21 days, then supplemented with 20 μl of MTS solution in 96 plates and incubated at 37˚C for 3 hours. 200 μl of supernatant was used to measure optical density spectrophotometrically at 490 nm (20, 22), using a microplate reader (Thermo, USA).

### Statistical analysis

Statistical significance was assessed using oneway analysis of variance (ANOVA), and the minimum significant difference between individual group means was calculated using the t test method. For a comparison of 2 groups, a 2-tailed unpaired student t test was used. Values of p less than 0.05 were considered significant. All data were reported as mean ± standard deviation (SD) (n=5).

## Results

### Histological comparison of intact and denuded HAMs

**Fig 1:**
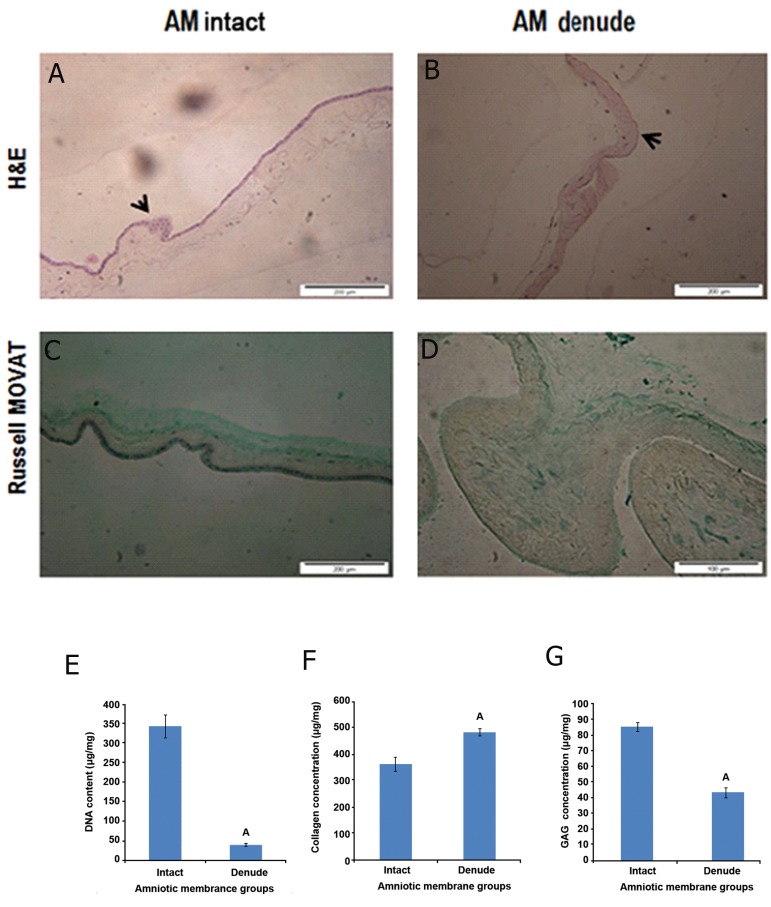
Decellularization of human amniotic membrane (HAM): hematoxylin- and eosin (H&E)-stained native HAM (original magnification: ×20) Intact HAM (A), 0.03% (w/v) sodium dodecyl sulphate (SDS)-treated HAM (original magnification: ×20) (B), in each image, the arrows are indicating the apical surface of the HAM. Extracellular matrix (ECM) compositions were showed in intact AM, dendued AM and 3D AM scaffold (C, D) by using Russell-Movat staining (collagen, yellow) and (GAG, Green), Deoxyribonucleic acid (DNA) content of intact and denuded HAM was quantified using a micro plate fluorescence reader (E). Statistical differences between intact and denuded HAM groups; analysis of ECM components, including acid/ pepsin-soluble collagen, sulfated GAG (F, G). Statistical differences between collagen and GAG contents of intact HAM and 3D AM scaffold. (Data are shown as mean ± standard deviation), n=5 , A; P<0.001 and GAG; Glycosaminoglycan.

Intact and denuded HAMs were stained using H&E and dyes to determine whether the treatment successfully eliminated cellular components. For routine histology, all samples were embedded using paraffin wax and sectioned and 5 sections at 6 μm were obtained and stained. H&E staining confirmed that the procedure was successful and no cells were visible (Fig 1A, B). Russell MOVAT staining demonstrated no obvious disruption to the sum of matrix histoarchitecture following treatment; the main structural component of HAM (collagen) appeared to have been preserved after decellularization (Fig 1C, D).

### Quantification of residual DNA following decellularization

The DNA content of HAM before treatment was determined as (341 ± 29.60 μg/ml). After the decellularization procedure, a significant decline to (39.38 ± 4.04 μg/ml) was observed (n=6, p<0.05, ANOVA, Fig 1E).

### Collagen and GAG analysis

Biochemical assays were undertaken to evaluate the ECM components after decellularization. The hydroxyproline content of intact AM was found to be (361 ± 27.39 μg/mg); after treatment, a significant increase to 478 ± 14.42 μg/mg (n=5, p<0.05, ANOVA) was observed (Fig 1F). GAGs form the major structural components of the ECM of tissues; their abundance in intact AM was found to be 85 ± 3.29 μg/mg. After treatment, a significant decrease to 43 ± 3.08 μg/mg (n=5, p<0.05, ANOVA) was observed (Fig 1G).

### Scaffold characteristics

**Fig 2:**
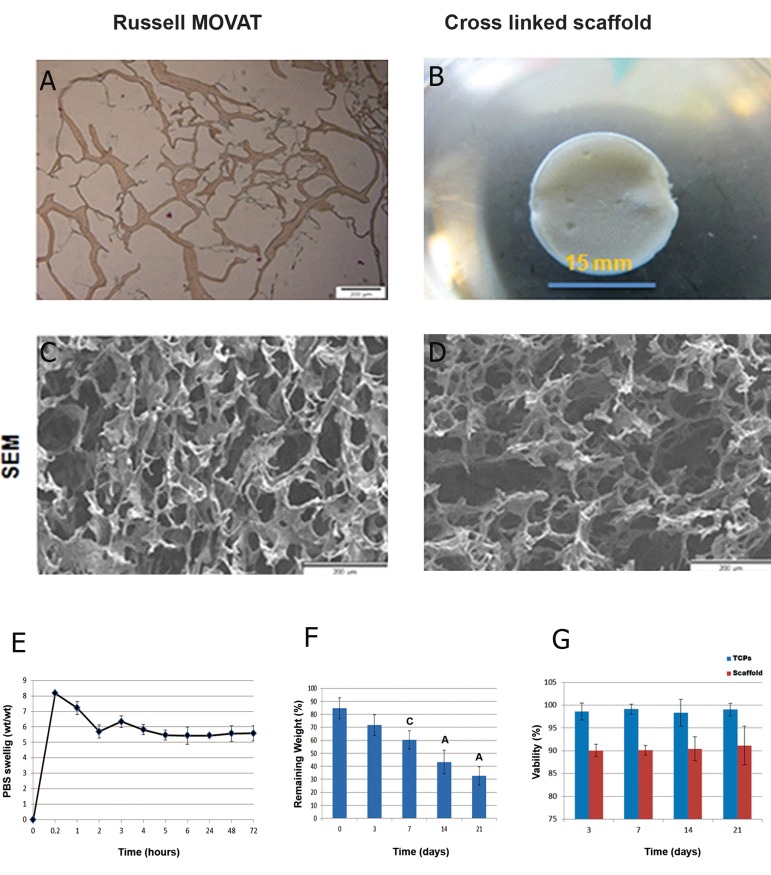
3D AM scaffold using Russell- Movat staining (collagen, yellow) and (GAG, Green) (A). Cross linked ECM derived AM scaffold produced by freeze dryer (B). SEM image of the surface (C). The cross section of the porous (D). PBS swelling ratio of ECM derived human AM scaffolds at different times (E). In vitro collagenase biodegradation; time course of weight remaining of ECM derived HAM scaffold, cross-linked with ratio (1:4) of NHS/EDC, after incubation in PBS containing 100 μg collagenase I, at 37˚C (F). Comparison results of effect of extract cytotoxicity of TCPs and scaffold groups on viability fetal fibroblast cells by MTS assay extract showed, (p>0.05) (G). (Data are shown as mean ± standard deviation). ECM; Extracellular matrix, AM; Amniotic membrane, GAG; Glycosaminoglycan, SEM; Scanning electronic microscopy, EDC; 1-ethyl-3-(3-dimethyl aminopropyl) carbodiimide hydrochloride, NHS; N-hydroxysuccinimide, PBS; Phosphate-buffered saline, TCP; Tissue culture plates, n=5, A; P<0.001 and C; P<0.05.

The main structural component of HAM (collagen) was showed by Russell MOVAT staining (Fig 2A). The thickness of 3D spongy scaffold in this study was about 4 mm to mimic the real thickness of human skin. The SEM observation results (Fig 2B) showed the morphological characteristics of the 3D spongy AM scaffolds. The scaffold disclosed extremely interconnected porous structures, and the pore wall surface appeared rough and homogeneous (Fig 2C, D). SEM images of cross-linked 3D spongy AM scaffolds indicated that it had an open porous structure with pores ranging from 44 to 160 μm. The mean pore size was 90 μm and the average porosity was 90%, that is suitable for cell penetration, nutrients and gas change.

### Cross-linking degree

Cross-linking of biological tissue materials using water-soluble carbodiimide has received much attention in the field of biomaterials science (24). Therefore, the 3D spongy AM scaffolds were cross-linked with EDC/NHS according to the general reaction mechanism. The results of the TNBS test showed that the crosslinking efficiency of AM derived ECM scaffolds was about (65% ± 10.53).

### PBS solution adsorption

We applied the swelling ratio test to assess water absorption capability and showed (Fig 2E) that without NHS/ EDC cross-linking, scaffolds dissolved in water within 2 minutes and couldnʼt maintain solid constructions. Our ECM components of 3D spongy AM scaffold cross-linked with NHS/ EDC presented a swelling ratio of approximately 5 fold compared with dry weight scaffold. The results showed highly increased swelling ratios at 5 minutes. Significant differences in swelling ratios were not observed at other selected time intervals (Fig 2E).

### In vitro collagenase degradation

The biological degradation of the 3D AM sponge-like scaffold was characterized by measuring the decrease in weight. The rates were tested by *in vitro* enzyme assays using collagenase I. Figure 2F shows that 100 μg/ml of collagenase I solution decomposed the scaffold gradually over three weeks. The scaffold was 29.344 ± 4.87% of the original weight after 21 days of treatment. *In vitro* enzyme biodegradations were evaluated to show the time dependences of this scaffold.

### Proliferation of cells directly in contact with scaffolds

The extract cytotoxicity assay distinguished the effect of soluble components of 3D spongy AM scaffold on the viability of primary human fetal dermal fibroblasts cells. Incubation of primary human fetal dermal fibroblasts with soluble extracts from intact AM, 3D spongy AM scaffold and tissue culture plate (TCP) displayed different levels of cell viability according to MTS assay. Extracts prepared from the 3D spongy AM scaffold, showed no significant difference in the viability of the fetal fibroblasts cells compared to the TCP group (cells-only negative control) and 3D spongy AM scaffold after 14 and 21 days (n=6, p>0.05, ANOVA). The extracts from the 3D spongy AM scaffold did not display significant adverse effects on the viability of the fetal fibroblasts cells (Fig 2G).

### Cell morphology

**Fig 3:**
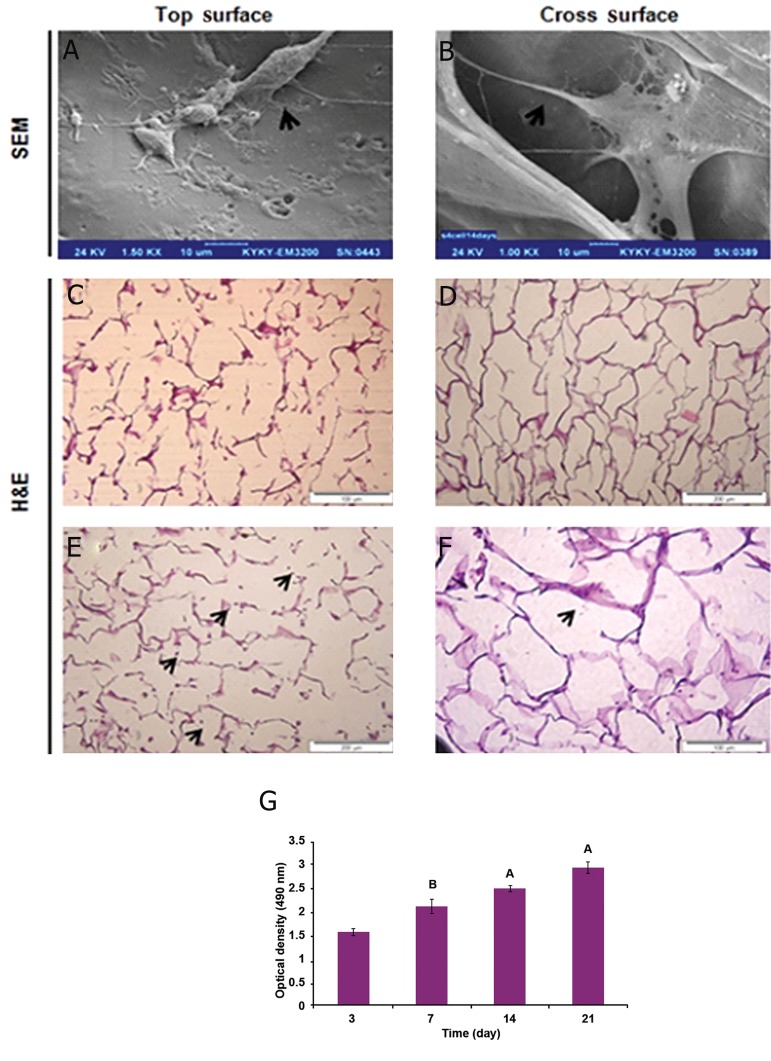
SEM images of fetal fibroblast cells attached (arrows are indicating fibroblast cells) to ECM derived HAM scaffolds, after 7 days at surface (A) and internal surfaces of 3D spongy scaffold (B) obtained by cross sectioning. H&E images before and after seeding cells, The light microscopy images of H&E images showed the external surface of scaffold without cell (C) and attachment of human fetal fibroblast cells at external surfaces of scaffold, the arrows are indicating attachment of fetal fibroblast cells, the cells are dark grey and the AM scaffolds are light red (D). H&E images show the internal surface of the scaffold without cell (E) attachment and growth of fetal fibroblast cells at internal surface of scaffold after 7 days (F). MTS results showed the metabolic activities of fetal fibroblast cells in ECM derived HAM scaffold. Statistical differences in metabolic activity at days 7, 14 and 21 with 3D HAM scaffold in days 3 (G). SEM; Scanning electronic microscopy, ECM; Extracellular matrix, HAM; Human amniotic membrane, H&E; Hematoxylin and eosin. (Data are shown as mean ± standard deviation (SD). (n=5, A; P<0.001 and B; P<0. 01).

The cell morphology of fibroblasts was studied on the scaffolds after 7 days of culturing. SEM images indicated fibroblast cells formed normal spindle-shaped cells on all scaffolds (Fig 3A, B). As shown H&E images of scaffold without cell (Fig 3C, D) and fibroblast cells were able to penetrate, attach and grow into the 3D structures of 3D spongy AM scaffold (Fig 3E, F) because of the presence of large pores.

### Cell metabolic activities in scaffolds

Cell metabolic activity of fetal fibroblast cells in 3D spongy AM scaffolds were evaluated at every indicated time interval based MTS assay (Fig 3G).The results of metabolic activity of human fetal fibroblast cells in 3D spongy AM scaffolds showed an increasing trend over 7, 14, and 21 days, but no significant differences were observed during 3 and 7 days of incubation.

## Discussion

AM is applied in surgery particularly for the reconstruction of traumatic wounds and skin transplantation (12). HAM is an appropriate substitute for general skin for surgical use due to its availability, low cost, and low risk of viral disease transmission and immunologic rejection. Basement membrane in human placenta-derived ECM could perform a functional component in the well regeneration of damaged basement membrane skin tissue, adjust fibroblast and keratinocyte development and differentiation, and construct epithelial tissue (12). For a logical design of scaffolds for skin engineering, it is fundamental to study the features and effect of individual components of biomaterial. The overall aim of this study was to develop an acellular matrix scaffold suitable for tissue engineering applications in the form of a 3D scaffold and as a cell delivery system (24). The decellularization procedure must eliminate the main sources of immunogenic response including cellular components, membrane antigens, and soluble proteins, so blocking initiation of immune response and later latest degradation of the acellular matrix transplanted in to the patient (17). A number of methods for the removal of cells from HAM have been investigated with varying degrees of success (25, 26). In most cases, when assessing cell removal and maintenance of matrix structure, the methods used failed to remove all of the cells and cellular components from the tissue matrix. In this experiment, the decellularization procedure of was accomplished according to a modified protocol that has been previously used on HAM (17).

The AM was decellularized by EDTA, SDS in two steps without the use of nuclease (DNAse and RNAse) unlike in other studies (17), and were impressive in terms of elimination of the cellular component. During the decellularization procedure in this study the hypotonic buffer lyses the cells by swelling the water in the cells and SDS, which is an ionic detergent, attaches to cell membranes and causes the destruction of the lipid bilayer. EDTA and the pH of the buffers blocked the activation of proteases during cell lysis (17). Results of the procedure to eliminate cells from HAM showed the loss of cells but retention of DNA in the matrix.

Results of the hydroxyproline assays (Fig 1F) indicated that the decellularization process did not lead to loss of collagen, elastin, or GAG content of the tissue. There was a statistically significant increase in all the structural components; this increase was probably as a result of extraction (by dry weight) of other soluble constituents (soluble proteins, lipids, nucleic acids).

Assessment of the hydroxyproline content using a collagen kit (Fig 1F) and Russel MOVAT staining, (Fig 1A, B), (Fig 2A) showed that the decellularization method did not lead to a decrease of the collagen contentin the AM. Collagen is an important component for cell proliferations and tissue body formation. It provides some of the mechanical properties such as adhesive and tensile strength. There was a statistically significant increase in this structural component of ECM compared to intact AM; the main reason for this increase maybe an elicitation of other soluble protein and lipids constituents.

Cultivation of cells in 2D monolayer cannot provide an adequate *in vivo* micro-environment for proliferation (26, 27). To fabricate an appropriate 3D scaffold in skin tissue engineering, various definitive factors to consider include pore size range, mechanical strength, biodegradability. AM dissolves because of endogenous enzymatic degradation of AM matrix during 1 week (28). For better use of AM in tissue engineering, it should be reinforced against enzymatic degradation. Collagen fibers constitute the main structure of AM which can easily undergo cross-linking, by bridges are made between the collagen chains (29, 30). Recently, EDC/NHS one of the cross-linker agents, has been utilized to improve mechanical properties in collagen (10), collagen-chitosan (11), and collagen-phosphorylcholine to obtain suitable tissue engineered corneal substitutes. NHS/EDC are presumed to be water-soluble and non-toxic crosslinking agents because they can be made from urea derivatives (15). Cross-linking has been confirmed to play a main role related to the porous structure distribution of the scaffold and water absorption. For this experiment, the 3D spongy AM scaffold was generated through lyophilization (Fig 2B). After cross-linking, this scaffold did not dissolve in water and was able to maintain its structure the culture medium. The swelling ratio results at selected time intervals disclosed that the scaffold possessed excellent porous lamellar matrix spaces which increased the water containing capacity. Because of the high water absorption feature, the sponge-like matrices were optimal for cells to culture in (27).

The degradation data presented gradual weight loss of the scaffold at selected time intervals (Fig 2F). Our scaffold was composed by NHS/EDC, was degraded by collagenase I and after it had decomposed; the scaffold lost its structural properties.

When constructing the skin graft, the establishment of the dermis over the model was apparently accelerated by the application of skin cells to the graft (28). Fibroblast cells perform active roles in a diversity of biological procedures such as the production of collagen, GAG and ECM proteins. In particular, fibroblast cells produce intra/extracellular cytoskeleton tension forces which allow for interaction with the ECM (29). SEM observations showed the fetal fibroblast cells seeded in the scaffold that they proliferated normally, confirming the benefit of these materials to cell growth (Fig 3A, B).

The interconnected pores within the scaffold provided the space status for interactions of biological cytokines and growth factors released from keratinocyte and fibroblast cells (30, 31).The resulting data from seeding fetal fibroblast cells on the scaffold was significant proliferation on the day 21compared to 3 day, which displayed that not only the cell proliferation was promoted, but the individual collagen constructing abilities were also enhanced (Fig 3G). As our scaffold has demonstrated the ability to increase collagen secretion, it is potentially a good biomaterial for wound healing in skin tissue engineering. Our 3D spongy AM scaffold hasexcellent potential because of its suitable pore size, the great swelling ratio and good cytocompatibility. The skin medicine and therapeutic wound dressing market is significant. Bio-functions of traditional dressings in the past are only for keeping the wound dry and preventing infection. In clinical applications, we know that moist and warm surroundings aid repair of wounds to the skin. Effective scaffolds must investigate several main factors including skin tissue evaluation s, tissue deficiency managements, humidity containing equilibrium, infection preventions, inflammation controls and dermatological wound edge progression enhancing in animal model. Other issues that need to be considered are; the patient healthy conditions (e.g. diabetes, burns), the injury type being created by physical or chemical damage, and the environmental properties. We will continue focusing on these important options about skin tissue engineering skin wound dressings in future studies.

## Conclusion

A diversity of biological scaffolds has been made with distinctive biochemical, biomechanical, and morphological properties. Different procedures may be used to fabricate organ-specific scaffolds for tissue engineering. In this study, HAM-derived ECM scaffolds composed of various ECM components were created as a biological scaffold for skin tissue engineering. Human ECM scaffolds were constructed from HAM via pulverization, decellularization, and lyophilization. We found that the sponge-like AM-derived ECM scaffold provided an optimal pore size and water absorption for human skin cell growth. This scaffold could be degraded by collagenase I, which demonstrates its biodegradability. Our results show that HAM-derived ECM scaffold could be useful in skin tissue engineering due to its physico-mechanical properties, which may improve the quality of wound healing.
